# Optimal Management of Patients with Moderate-to-Severe Inflammatory Bowel Disease

**DOI:** 10.3390/jcm13237026

**Published:** 2024-11-21

**Authors:** Tugrul Purnak, Atilla Ertan

**Affiliations:** Division of Gastroenterology, Department of Internal Medicine, Faculty of Medicine, University Texas McGovern Medical School, Houston, TX 77030, USA; atilla.ertan@uth.tmc.edu

**Keywords:** inflammatory bowel disease, biologic therapies, immunomodulators, dulators Janus kinase (JAK) inhibitors

## Abstract

Inflammatory bowel disease (IBD), encompassing Crohn’s disease (CD) and ulcerative colitis (UC), is a chronic and often debilitating condition requiring complex and individualized management. Over the past few decades, advancements in understanding IBD pathophysiology have led to a transformative shift in therapeutic approaches. This article provides a comprehensive overview of the evolution of IBD treatments, from early symptom-focused therapies to modern biologics, small molecule agents, and emerging treatment strategies. We discuss therapeutic goals centered on achieving clinical remission, endoscopic/mucosal healing, and enhancing patient quality of life. Additionally, we explore the rationale for the early and personalized use of biologic therapies in moderate-to-severe cases, review the current FDA-approved agents as of 2024, and highlight the advantages and limitations of these treatments. Special attention is given to the evolving role of novel oral therapies, including Janus kinase inhibitors and sphingosine-1-phosphate receptor modulators, and future new directions. This paper aims to guide clinicians in navigating the expanding therapeutic landscape of IBD, emphasizing patient-centered decision-making and addressing ongoing challenges in achieving optimal disease control.

## 1. Introduction

Inflammatory bowel disease (IBD), which includes Crohn’s disease (CD) and ulcerative colitis (UC), represents a chronic and challenging condition that necessitates intricate and tailored treatment strategies. Advances in our understanding of IBD pathophysiology over recent decades have profoundly altered therapeutic paradigms. This paper examines the progression of IBD treatment approaches, tracing the shift from symptom-based therapies to modern options, such as biologics, small molecules, and other emerging strategies. We discuss key therapeutic objectives, such as achieving clinical remission, promoting endoscopic/mucosal healing, and improving quality of life for patients. The article also addresses the importance of the timely, individualized use of biologics in moderate-to-severe cases, reviews FDA-approved therapies available in 2024, and considers their respective benefits and drawbacks. Particular focus is placed on the growing role of innovative oral therapies, including Janus kinase inhibitors and sphingosine-1-phosphate receptor modulators, while future new possibilities are also considered. This article aims to support clinicians as they navigate the evolving landscape of IBD treatment, promoting patient-centered choices and addressing persistent obstacles in achieving optimal disease management.

### 1.1. Historical Evolution and Advancements in IBD Treatment

Inflammatory bowel disease (IBD), which encompasses both Crohn’s disease (CD) and ulcerative colitis (UC), has a lengthy and complex history of treatment. Table 1 presents a chronological overview of the most significant developments in the history of IBD treatment, including the introduction of pivotal drugs and therapeutic approaches over time. The initial therapeutic options were primarily directed towards symptom management rather than disease modification. Corticosteroids, such as prednisone, were primarily utilized due to their potent anti-inflammatory effects [1]. However, the long-term use of steroids was associated with adverse effects, including osteoporosis, steroid-induced diabetes, and an increased risk of infection. In addition to steroids, 5-aminosalicylic acids (5-ASAs), including sulfasalazine and mesalamine, constituted the primary means of managing mild-to-moderate UC. However, they proved ineffective in maintaining CD.

In the 1980s, purine analogs, initially developed for the prevention of organ transplant rejection, were identified as a potential treatment for IBD. Drugs such as azathioprine and 6-mercaptopurine (6-MP) were subsequently employed extensively in maintenance therapy, particularly in the case of CD. They proved effective in preventing relapses and reducing the necessity for steroids. However, the most significant concern regarding these drugs is their adverse effects, particularly the development of lymphoma [2].

Another notable advancement was the introduction of methotrexate, an immunomodulatory agent that functions by inhibiting dihydrofolate reductase, thereby suppressing the immune system. Methotrexate was identified as a potential alternative for patients who did not respond to or could not tolerate azathioprine or 6-MP [3].

The late 1990s saw a significant advancement in the treatment of IBD with the advent of biologic therapies. In 1998, infliximab became the first biologic agent to be approved for the treatment of IBD. It targets tumor necrosis factor-alpha (TNF-α), a cytokine involved in systemic inflammation, and provides a more targeted approach, significantly improving outcomes for some patients with moderate-to-severe CD and UC who were refractory to conventional therapies. Subsequently, other anti-TNF agents, including adalimumab, certolizumab pegol, and golimumab, were incorporated into the therapeutic armamentarium [4].

As knowledge of the immune pathways involved in IBD expanded, new biologics targeting different molecules were developed. These included vedolizumab, an integrin inhibitor that selectively blocks lymphocyte trafficking to the gut, and ustekinumab, which inhibits interleukin -IL-12 and IL-23, cytokines involved in the inflammatory process. The advent of biosimilars—biologic products that are highly similar to existing biologics—has resulted in a reduction in treatment costs due to their lower price point. Biosimilars such as infliximab-dyyb and adalimumab-atto have been approved for use in IBD, offering cost-effective alternatives that do not compromise efficacy [5].

In recent years, small molecule therapies, such as Janus kinase (JAK) inhibitors and sphingosine-1-phosphate ( S1P) receptor modulators, have emerged as important options for patients who are intolerant to or have failed biologic therapies. These agents offer the convenience of oral administration and expand the therapeutic options for managing IBD. This evolution in treatment reflects a deeper understanding of IBD pathophysiology, which has led to the development of more targeted, effective, and less costly therapies. As a result, what was once a debilitating condition has become a manageable disease with a range of therapeutic options [6].

### 1.2. Therapeutic Goals in IBD

The management of IBD has undergone a notable evolution, shifting from a primary focus on symptom control to a more comprehensive strategy aimed at achieving deep and sustained clinical remission and even histologic healing. The primary therapeutic goals now encompass both clinical and endoscopic outcomes, with the objective of improving long-term disease control and patient quality of life [7,8,9].

The primary objective in the management of inflammatory bowel disease (IBD) is to achieve clinical remission, which is defined as the absence of symptoms such as abdominal pain, diarrhea, and rectal bleeding. Achieving remission frequently necessitates the administration of induction therapy with corticosteroids, biologics, or small molecules, depending on the severity of the disease. In cases of mild disease, 5-ASA agents may prove sufficient; however, in instances of moderate-to-severe disease, biologics or small molecule immunomodulators are often required.

Once remission is achieved, it is of the utmost importance to prioritize proper and long-term maintenance management. The objective of maintenance therapy is to prevent the occurrence of flare-ups and disease progression, thereby reducing the risk of complications such as strictures, fistulas, the necessity for surgical intervention, and dysplastic changes.

Based on an extensive review of the existing literature, our extensive experience, and despite extensive research with the development of various agents for the treatment of IBD, the overall clinical response and mucosal/histologic healing rates remain modest. Approximately 50–60% of patients achieve a clinical response, while clinical remission is attained in only about 30–35% of cases. These figures underscore the ongoing challenges in IBD management, emphasizing the necessity for continued research and the development of more efficacious therapies [10].

### 1.3. Management of Moderate-to-Severe IBD Patients

Moderate UC and CD present with more frequent symptoms and moderate inflammation but generally lack severe systemic impact. Patients with moderate UC may have up to six bloody stools per day with mild abdominal pain, while moderate CD often involves intermittent pain, diarrhea, and minimal weight loss, with only mild elevation in inflammatory markers. Severe UC and CD are characterized by frequent, intense symptoms and significant systemic involvement. In severe UC, patients experience more than six bloody stools daily, intense abdominal pain, and often deep ulcerations throughout the colon. Severe CD is marked by intense abdominal pain, significant weight loss, and complications such as strictures or fistulas, reflecting deep ulcerations, widespread inflammation often involving multiple GI segments. Severe cases in both conditions typically present with markedly elevated inflammatory markers and profound fatigue, anemia, and hypoalbuminemia. The specific scores or criteria used to stratify patients are beyond the scope of this review; however, patients are generally categorized based on these clinical, endoscopic, and biochemical characteristics [11,12].

The management of patients with moderate-to-severe IBD necessitates a more assertive and personalized approach, given the complexity and severity of the disease. A principal objective of treatment strategies is to control inflammation, prevent complications, and improve long-term positive outcomes through early and intensive therapy. For patients with moderate-to-severe IBD, the early introduction of biologics, such as anti-TNF agents, vedolizumab, ustekinumab or IL-23 inhibitors are often recommended [12,13,14]. A substantial body of evidence, derived from extensive experience and rigorous scientific studies, has demonstrated that the early introduction of biologics, particularly in patients with poor prognostic factors, can result in superior outcomes, including higher rates of remission, mucosal healing, and a reduced need for surgical intervention.

The top-down approach entails initiating biologic therapy as the primary treatment option, rather than reserving it for patients who have not responded to conventional therapies. This strategy is particularly advantageous for patients with aggressive disease phenotypes, such as young patients with extensive CD or those with severe UC. Specific patient characteristics render the top-down approach an especially suitable course of action. Table 2 provides a summary of these criteria. The following table enumerates specific criteria that suggest a more aggressive approach to IBD treatment, thereby elucidating the rationale for early intervention in these patient groups [15].

In cases where a patient does not respond to or loses response to a specific biologic, switching to a different biologic with a distinct mechanism of action (e.g., from an anti-TNF to an integrin inhibitor, IL-12/23 inhibitor, or IL-23 inhibitor) may prove an effective course of action. Small molecule immunomodulator therapies, such as JAK inhibitors or S1P modulators, provide additional options for younger patients with IBD who have not responded to biologics. These agents provide a distinct mechanism of action and may prove efficacious in patients with refractory disease. In certain instances, surgical intervention may be imperative and should not be postponed for patients with intractable disease, particularly those with complications such as strictures, fistulas, or abscesses. It should be noted that surgical intervention is not a curative measure; however, it can be an integral component of a comprehensive management strategy for select patients with CD, particularly those with Crohn’s ileitis.

### 1.4. FDA-Approved Agents in 2024

As of 2024, the landscape of treatments for IBD has expanded significantly, with a wide range of drugs approved by the Food and Drug Administration (FDA) offering targeted, individualized treatment options for patients with varying disease phenotypes and severities. Table 3 presents a list of medications that have been approved by the FDA for the treatment of IBD.

## 2. Biologic Therapies

### 2.1. Anti-TNF Agents

*Infliximab* is a chimeric monoclonal antibody targeting TNF-α that has been approved for both CD and UC. This has been a transformative treatment for patients with moderate-to-severe disease [16].

Infliximab was the inaugural biologic therapy to be approved for the treatment of CD. It is a chimeric antibody, derived from mice, administered intravenously over a period of 2–3 h. The initial treatment regimen comprises three infusions over a six-week period, followed by maintenance doses every two months in the event of significant improvement [16,17].

Although infliximab is generally well tolerated, infusion reactions have been documented on rare occasions. Given the pivotal role of tumor necrosis factor (TNF) in the immune system, the use of infliximab and analogous biologics may elevate the risk of infections such as tuberculosis and hepatitis B. Consequently, patients must undergo screening for these infections prior to initiating treatment, and live vaccines should be avoided throughout the course of therapy [17].

*Adalimumab* is a fully human monoclonal antibody against TNF-α which has also been approved for both CD and UC. Adalimumab provides the advantage of subcutaneous administration, which is more convenient for patients than intravenous infliximab [18].

Following an initial loading dose, administered in two injections over a period of two weeks, the maintenance dose is administered every other week [19]. This medication is generally well tolerated, with the most frequent adverse effect being localized cutaneous reactions at the injection site, such as pruritus, erythema, or edema. Adalimumab may also be an effective alternative for individuals who have not responded to or are unable to tolerate infliximab [10].

The secondary loss of response with both infliximab and adalimumab is very important due to immunogenicity that varies between 35 to 70% depemdent on the length of maintenance therapy.

*Certolizumab pegol* is a subcutaneous and pegylated humanized anti-TNF antibody fragment that has been approved for the treatment of CD. Pegylation serves to diminish the immunogenicity of the drug and extend its half-life [20].

In 2008, the United States Food and Drug Administration (FDA) approved the use of this medication for the treatment of moderate-to-severe CD in patients who have not responded adequately to conventional therapies. Certolizumab is administered via subcutaneous injection, with two doses administered over a two-week period, followed by monthly injections for maintenance. It has been demonstrated to be an efficacious treatment for inducing and maintaining remission in CD, with a comparable safety profile to that of infliximab and adalimumab. In some patients who are unable to tolerate other TNF alpha agents, this medication can be effective [10].

*Golimumab* is approved for UC; golimumab is another subcutaneous anti-TNF agent that offers flexibility in treatment regimens [21].

The main side effects include nasopharyngitis, pharyngitis, laryngitis, rhinitis, and tuberculosis reactivation [22].

### 2.2. Biosimilars

The advent of biosimilars for infliximab (e.g., infliximab-dyyb and infliximab-abda) and adalimumab (e.g., adalimumab-atto) has facilitated broader access to biologic therapies, offering cost-effective alternatives that do not compromise efficacy or safety. The availability of these agents is contributing to a reduction in healthcare costs and an increase in treatment accessibility for a broader patient population.

These FDA-approved agents provide clinicians with a toolkit that allows them to customize treatment plans to the specific needs and circumstances of each patient. Biosimilars are supported by the majority of insurance companies in Europe and, recently, in the United States. The Biologic Price Competition and Innovation Act was enacted as part of the Affordable Care Act in 2010 [23,24,25].

### 2.3. Integrin Inhibitors

*Natalizumab*, an α4 integrin inhibitor, was initially approved by the FDA for the treatment of CD. Subsequently, this very effective drug was withdrawn from the market due to an increased risk of progressive multifocal leukoencephalopathy [26]. The half-life of natalizumab is prolonged, with the maximum concentration achieved one to two hours following intravenous administration. The terminal half-life is between 10 and 11 days, necessitating administration every four weeks [22].

*Vedolizumab* is an α4β7 integrin inhibitor that selectively targets the gut, reducing inflammation without the adverse effects associated with systemic immunosuppression. This relatively most safest biologic agent has been approved for both CD and UC and has been shown to be particularly beneficial for patients at high risk of infection. Although there is a theoretical risk of progressive multifocal leukoencephalopathy (PML) due to its potential effect on lymphocyte trafficking to the brain, there have been no reported cases of PML associated with its use at this time [27].

Although vedolizumab is slower to take effect than natalizumab, peak levels are reached within 1–2 h after infusion. The terminal half-life is considerably longer, at approximately 25 days, thereby enabling administration at 8-week intervals [13,28].

### 2.4. Interleukin Inhibitors

*Ustekinumab* targets interleukin-12 and interleukin-23, which are cytokines that are involved in the immune response. It has been approved for both CD and UC, offering a novel mechanism of action for patients who have not responded to anti-tumor necrosis factor (anti-TNF) therapy [29]. Ustekinumab is a fully human IgG1k monoclonal antibody that binds to the p40 subunit of IL-12/23. It inhibits the binding of IL-12 and IL-23 to IL-12Rb1, thereby preventing the initiation of downstream signaling cascades and the production of cytokines. The U.S. Food and Drug Administration (FDA) has granted approval for ustekinumab for the treatment of psoriasis, Crohn’s disease, and ulcerative colitis.

Ustekinumab is administered intravenously (IV) for induction therapy at a dose of 6 mg/kg, followed by maintenance with 90 mg subcutaneously (SC) every 8 weeks.

In patients with refractory CD, ustekinumab demonstrated a clinical response rate of 49% at week 22, which was significantly higher than the 27% observed in the placebo group [29,30].

Following the induction phase, maintenance therapy resulted in 60% of patients achieving clinical remission at one year and 51% maintaining remission after five years [28]. Furthermore, ustekinumab exhibited superior efficacy compared to vedolizumab in patients with refractory CD. However, the healing of perianal fistulas with this very expensive biologic agent was limited to 28% [31].

In patients with moderate-to-severe UC, ustekinumab demonstrated superior efficacy compared to placebo for both induction and maintenance therapy, with response rates of 62% and 31%, respectively [14].

Rare but serious side effects include reversible posterior leukoencephalopathy syndrome (RPLS), characterized by symptoms such as headache, confusion, and vision changes, along with an increased risk of infections [30,32]

*Risankizumab* and *Mirikizumab* are humanized monoclonal antibodies that selectively target the p19 subunit of IL-23, thereby inhibiting the production of IL-17, TNF-alpha, IL-21, and INF-gamma.

The phase 3 ADVANCE and MOTIVATE trials were conducted to assess the efficacy of risankizumab as an induction therapy for patients with moderately to severely active CD. Patients were administered either 600 mg or 1200 mg of intravenous risankizumab or placebo at weeks 0, 4, and 8. The results of the trials demonstrated that both doses of risankizumab resulted in a significantly greater improvement in clinical remission and endoscopic response compared to placebo at week 12. In the ADVANCE trial, the clinical remission rates were 45% with 600 mg of risankizumab and 42% with 1200 mg, in comparison to 25% with placebo. Endoscopic response rates were 40% and 32% with 600 mg and 1200 mg of risankizumab, respectively, in comparison to 12% with placebo. The results of the MOTIVATE trial were comparable, with risankizumab demonstrating consistent efficacy in patients who had previously failed to respond to other biologic therapies. Risankizumab was generally well tolerated across both trials [33].

In a head-to-head clinical trial comparing risankizumab and ustekinumab in patients with moderate-to-severe CD who had previously failed anti-TNF therapy, risankizumab demonstrated superior efficacy. By week 24, 58.6% of patients treated with risankizumab achieved clinical remission, compared to 39.5% of those treated with ustekinumab. At week 48, risankizumab was also more efficacious in achieving endoscopic remission, with 31.8% of patients exhibiting improvement versus 16.2% in the ustekinumab group. Both drugs exhibited comparable safety profiles, although risankizumab demonstrated a slightly lower incidence of serious adverse events. In conclusion, risankizumab proved to be a more efficacious agent for inducing and maintaining remission in CD [33,34,35,36].

The main side effects of risankizumab include upper respiratory tract infections, headaches, and injection site reactions. Serious adverse events, though rare, may include severe infections and hypersensitivity reactions, and there is an observed risk of malignanciessuch as cutaneous basal and squamous cell carcinoma [37].

Mirikizumab was administered with an intravenous loading dose of 300 mg at weeks 0, 4, and 8, followed by a subcutaneous dose of 200 mg every 4 weeks. In patients with moderately to severely active ulcerative colitis, the clinical remission rate was 24.2% at week 12, compared to 13.3% with placebo. At week 40, the remission rate was 49.9% with the drug and 25.1% with placebo for maintenance therapy. Additionally, the drug demonstrated favorable outcomes in terms of endoscopic remission and bowel urgency, with a low incidence of adverse events, including headache and nasopharyngitis [38].

Both agents demonstrated efficacy in patients who had previously failed therapy targeting tumor necrosis factor (TNF) as well as in patients who had not yet received TNF-targeting therapy. The incidence of adverse events was low, with the most commonly reported being headache, arthralgia, upper respiratory tract infections, and hepatotoxicity [33,34,35,36].

*Guselkumab* is an interleukin-23p19 subunit antagonist that works by inhibiting the binding of IL-23to its receptor on the cell surface. IL-23 plays a critical role in the pathogenesis of inflammatory bowel diseases like UC by promoting cytokine production and immune system activation. By blocking this interaction, guselkumab helps reduce inflammation and the immune response in the gut, which is beneficial for patients with moderate-to-severe UC. Recently, the FDA approved guselkumab for the treatment of moderately to severely active ulcerative colitis.

The QUASAR phase 2b study evaluated the efficacy and safety of guselkumab, in patients with moderately to severely active UC. The double-blind, placebo-controlled trial randomized patients to receive either 200 or 400 mg of intravenous guselkumab or placebo. The primary endpoint, clinical response at 12 weeks, was significantly higher in the guselkumab groups compared to placebo, with improvements also observed in clinical remission, symptomatic remission, endoscopic improvement, and histo-endoscopic mucosal healing. Safety profiles were comparable across all groups, and guselkumab showed promise as a safe and effective treatment for UC [39].

The VEGA study was a randomized, double-blind, phase 2 trial aimed at assessing the efficacy of combination therapy with guselkumab and golimumab compared to monotherapy with either drug in patients with moderately to severely active ulcerative colitis. The trial involved 214 participants and measured clinical response at 12 weeks, with results showing that 83% of patients in the combination group achieved clinical response compared to 61% and 75% in the monotherapy groups. While the combination therapy demonstrated promising efficacy, it did not meet the predefined statistical significance criteria for clinical remission compared to guselkumab alone. The most common adverse events included ulcerative colitis exacerbation, upper respiratory infections, and headaches, with no deaths or cases of malignancy reported during the induction period. The findings suggest that guselkumab and golimumab combination therapy may provide enhanced clinical responses in UC treatment, warranting further investigation in larger studies [40].

The DUET-CD and DUET-UC trials are ongoing phase 2b studies that are randomized, double-blind, and placebo-controlled, involving patients with moderate-to-severe active CD and UC, respectively. Participants are assigned to one of six groups, which include guselkumab, golimumab, and JNJ-78934804 (a combination of guselkumab and golimumab) at high, mid, and low doses, as well as a placebo group. The primary objective is to evaluate clinical remission and endoscopic response across treatment groups at week 48 [41,42].

Recently, the FDA approved guselkumab for the treatment of moderately to severely active UC. Active and promising trials are pending in patients with moderate-to severe CD.

## 3. Oral Small Molecule Therapies

### 3.1. Janus Kinase (JAK) Inhibitors

*Tofacitinib* is an oral, rapidly acting Janus kinase (JAK) inhibitor that primarily targets JAK1 and JAK3, and which has been approved for the treatment of UC. It provides an alternative to biologics for patients who have failed to respond to other treatments, particularly for those who prefer oral administration. However, its use necessitates meticulous observation due to the potential for adverse effects, including thromboembolism and an elevated risk of herpes zoster infection [43,44].

Phase 3 studies (Octave) demonstrated the efficacy of the treatment in achieving higher rates of clinical remission in patients with UC, both in the induction (8 weeks) and maintenance (52 weeks) trials [45]. A meta-analysis substantiated the efficacy of the treatment in patients with refractory UC s while also demonstrating a favorable safety profile [46]. A comparative analysis of tofacitinib and vedolizumab in patients with UC who had failed to respond to anti-TNF therapy revealed that tofacitinib demonstrated superior efficacy with comparable safety profiles, thereby establishing it as a promising therapeutic alternative for UC [47].

A recent meta-analysis revealed that tofacitinib markedly enhanced health-related quality of life across a range of domains, including bowel symptoms (diarrhea, abdominal discomfort), systemic symptoms (fatigue), emotional function (decreased anxiety, depression), and social function (enhanced participation in daily activities). Tofacitinib demonstrated the greatest efficacy in improving health-related quality of life during the maintenance phase, underscoring its capacity to sustain long-term quality of life enhancements for patients with UC [48]. However, over 65 years patients who already have an increased cardiovascular diseases, especially smokers, tofacitinib is not a safe treatment option. *Upadacitinib* is a selective JAK1 inhibitor with a relatively favorable adverse event profile. The drug has demonstrated efficacy in both CD and UC [49]. Sandborn et al. demonstrated that upadacitinib was more efficacious than a placebo in patients with UC during the induction period. However, the study also revealed that upadacitinib induced elevations in serum lipid levels and creatine phosphokinase [44,50].

In a phase 3 study, the group receiving upadacitinib demonstrated significantly superior clinical improvement compared to the placebo group, both during the induction and maintenance phases in patients with moderate-to-severe UC [51].

The most commonly reported adverse effects were increased creatine phosphokinase levels -not clinically significant-, nasopharyngitis, and acne [44,51].

A recent multicenter retrospective study, which included 31 patients diagnosed with UC or CD, demonstrated that treatment with upadacitinib following prior tofacitinib therapy resulted in substantial clinical improvements. In patients with UC, upadacitinib not only provided substantial symptom relief but also resulted in a notable reduction in inflammatory markers. These findings underscore the efficacy of upadacitinib as a viable treatment option for UC, particularly in instances where tofacitinib proved to be either ineffective or poorly tolerated [52].

A recent meta-analysis has compared the efficacy of various biologics and small molecule therapies in the treatment of UC by focusing on patient-reported outcomes and health-related quality of life. The data from the 54 studies demonstrated that upadacitinib exhibited the highest efficacy in achieving clinical remission based on patient-reported outcomes, including stool frequency and rectal bleeding, during both the induction and maintenance phases [48].

The use of JAK inhibitors, such as upadacitinib and tofacitinib, may be beneficial for treating refractory ulcerative colitis (UC) and acute severe UC in patients unresponsive to standard treatments and biologics. A recent study demonstrated successful remission with upadacitinib following multiple treatment failures, highlighting JAK inhibitors as promising options for achieving remission in challenging cases [53].

### 3.2. Sphingosine-1-Phosphate (S1P) Receptor Modulators

*Ozanimod* is an oral agonist of the S1P1 and S1P5 receptors and has been approved for the treatment of UC. The drug reduces lymphocyte trafficking to the gut, thereby decreasing inflammation. The oral administration of the drug and its favorable safety profile make it a compelling option for especially younger patients with UC. Ongoing research trials are currently underway to assess the efficacy of this treatment in patients with moderate-to-severe CD [44,54,55,56].

Given the involvement of S1P signaling in cardiovascular functions, the use of S1PR1 modulators such as ozanimod may potentially result in the occurrence of cardiac side effects, including bradycardia and atrioventricular block [57].. Ozanimod has been demonstrated to be an efficacious treatment for moderate-to-severe UC, both during the induction phase (8–10 weeks) and the maintenance phase (24–52 weeks). This resulted in a higher rate of clinical remission compared to the placebo [54,58]. The most commonly observed adverse effects were mild elevations in liver enzymes, respiratory infections, and headaches. However, these were generally not severe enough to result in treatment discontinuation. In conclusion, ozanimod is regarded as an efficacious and well-tolerated therapeutic option for the management of UC [58,59].

A recent analysis demonstrated the efficacy of ozanimod in the treatment of patients with moderate-to-severe UC who had not yet received advanced therapies, including biologics and Janus kinase inhibitors. Improvements were observed as early as week 2, with significant differences evident by week 4. By week 10, 23% of patients receiving ozanimod had achieved clinical remission, compared to just 6.6% of those receiving the placebo. At week 52, 41.4% of patients remained in remission, and this continued into the open-label extension, where 91% maintained a clinical response through week 94. The safety profile was comparable to that observed in previous studies, with mild infections and elevations in liver enzymes being the most frequently reported adverse effects. In conclusion, ozanimod demonstrated a robust and sustained efficacy in these patients, establishing it as a promising early intervention before the introduction of more advanced therapeutic modalities [60].

*Etrasimod* is a S1P1 receptor modulator that offers comparable advantages to ozanimod and may provide an additional efficacious oral alternative for patients’ management with UC. Clinical trials are currently underway to evaluate the efficacy and safety of this treatment in patients with moderate-to-severe CD [61].

In the phase 2 Oasis trial, treatment with 2 mg of etrasimod resulted in significantly greater improvements in modified Mayo Clinic scores compared to placebo after 12 weeks, with only mild adverse effects reported [46]. Furthermore, the open-label extension of the Oasis study, conducted over a period of 52 weeks, corroborated the favorable safety profile of 2 mg of etrasimod in patients with UC [61].

The ELEVATE trials built upon the findings of the earlier Oasis trial by providing long-term data on the efficacy and safety of etrasimod in the treatment of moderate-to-severe UC. While the Oasis trial demonstrated short-term benefits over a 12-week period, the ELEVATE trials evaluated both the induction and maintenance phases. The ELEVATE UC 52 trial, in particular, extended to 52 weeks and demonstrated sustained clinical remission. Furthermore, detailed endpoints, such as endoscopic and histological improvement, were assessed, thereby underscoring etrasimod’s capacity to maintain remission and its favorable safety profile in a more expansive patient population [62].

## 4. Choice of First IBD Therapy

The selection of the initial therapeutic intervention in IBD represents a pivotal decision that has the potential to markedly influence the disease trajectory and overall quality of life of the patient. A reliable serologic biomarker for IBD patients is currently lacking. In light of the aforementioned considerations, it is not surprising that this is a frequently posed question by the community of gastroenterologists seeking guidance on the optimal initial treatment option. The decision-making process is influenced by a number of factors, including the severity of the disease, the location and extent of the inflammation, the patient’s comorbidities, and the presence of extraintestinal manifestations. In practice, we utilize IBD disease modifiers and the corresponding recommended first-line treatment options, as predicted in Table 4. Furthermore, patient preferences, potential adverse effects, and cost are additional factors that contribute to the determination of the most appropriate initial management strategy [12,44,63,64].

### 4.1. New Oral Agents in Development

The landscape of IBD treatment has undergone a notable transformation with the advent of new oral agents, offering more efficacious and patient-centric alternatives. These innovative therapeutic agents are designed to target specific pathogenic pathways involved in the development of IBD. They offer distinct advantages over traditional treatments, particularly for patients who prefer oral administration or are intolerant to injectable therapies.

### 4.2. Janus Kinase (JAK) Inhibitors

*Itacitinib* is a selective JAK1 inhibitor that has demonstrated potential for future therapeutic applications in the treatment of immune-mediated diseases. The clinical trials investigating the efficacy of this agent in patients with IBD are still ongoing [44].

*Ritlecitinib* and *brepocitinib* are oral inhibitors targeting JAK3/TEC and TYK2/JAK1, respectively. Both have demonstrated efficacy in the treatment of immune-mediated inflammatory conditions and are currently being investigated for their potential use in the management of IBD.

A phase 2b randomized, double-blind, placebo-controlled study was conducted to evaluate the efficacy and safety of these two oral agents as induction therapies over an eight-week period. Both ritlecitinib and brepocitinib demonstrated a dose-dependent improvement in total Mayo scores, with higher doses resulting in a greater reduction compared to the placebo. The primary endpoints, including clinical remission, endoscopic improvement, and histologic remission, demonstrated statistically significant positive outcomes with both drugs. Furthermore, the study demonstrated that both agents were well tolerated, with the majority of adverse events being mild or moderate. Infections were the most commonly observed adverse event, though no cases of herpes zoster were reported to be serious. In conclusion, both drugs demonstrated promising efficacy and acceptable short-term safety profiles. However, further long-term studies are necessary to fully understand their benefit–risk profiles for the management of UC patients [65].

*Deucravacitinib* is a novel orally administered TYK2 inhibitor that selectively targets the TYK2 enzyme without significantly affecting JAK1-3.

The phase 2 LATTICE-UC study was conducted to evaluate the efficacy and safety of deucravacitinib in patients with moderately to severe UC who had an inadequate or lost response to previous therapies. In this 12-week, double-blind study, 131 patients were randomly assigned to receive either deucravacitinib (6 mg twice daily) or placebo. The primary endpoint was clinical remission, yet the study did not achieve its primary or secondary endpoints. Notably, in patients who had previously undergone biologic therapy, clinical remission rates were higher with deucravacitinib (16.1%) than with the placebo (0%). Notwithstanding the numerical enhancements in symptomatic Mayo scores, the results remained statistically insignificant. The majority of adverse events were mild to moderate, and further trials are required to investigate the potential for higher doses [66].

### 4.3. Sphingosine-1-Phosphate (S1P) Receptor Modulators

*Amiselimod* is an oral selective modulator of the S1P1 receptor that has demonstrated promising results in the treatment of UC, with a rapid onset of action and only mild side effects. Amiselimod has demonstrated efficacy in reducing T-helper cell infiltration in chronic colitis models, thereby suggesting its potential as a treatment option for UC.

The objective of the phase II study was to assess the safety and efficacy of Amiselimod in patients with moderate-to-severe CD. Over a 14-week period, 78 participants were randomly assigned to receive either the investigational drug, Amiselimod (0.4 mg), or a placebo. The primary objective was to achieve a 100-point reduction in the Crohn’s Disease Activity Index (CDAI) by week 12. Although Amiselimod was generally well tolerated, with the majority of adverse effects being mild to moderate, it did not demonstrate superior efficacy compared to the placebo in achieving clinical response or remission. No new safety concerns were identified during the course of the trial [67].

### 4.4. Oral Integrin Inhibitors

The migration of lymphocytes to the gut mucosa represents a pivotal element in the pathogenesis of IBD. Chemokines and selectins play a pivotal role in this process, facilitating the adhesion of T cells to endothelial cells. Recently, novel small molecule oral therapeutic agents for the management of IBD patients have been developed that specifically target adhesion molecules. Examples of such agents include AJM300, PN-943, and MORF-057.

*AJM300* is a gut-restricted antagonist of the α4 integrin subunit that prevents the binding of α4β7 and α4β1 integrins on T cells to adhesion molecules, thereby inhibiting lymphocyte migration into the gut. Although AJM300 theoretically carries a risk of PML due to its potential impact on lymphocyte trafficking to the brain, there have been no documented cases of PML associated with its use to date. In light of the currently available evidence, AJM300 appears to be a promising new treatment option for inducing remission in patients with moderately active UC, pending further studies with long-term results [44].

*PN-943* is a small molecule that functions as an integrin antagonist by blocking the α4β7 receptor, with its effects largely confined to the gut. PN-943 is an oral small molecule that functions as an integrin antagonist by blocking the α4β7 receptor, with its effects largely confined to the gut. In a recent phase 2 study, which was double-blind, placebo-controlled, and conducted across multiple centers over 12 weeks, PN-943 demonstrated superior clinical remission rates compared to placebo in patients with ulcerative colitis, while exhibiting minimal adverse effects. A phase 3 trial for this promising oral therapy has yet to be conducted. [44,68].

*MORF-057* is another oral small molecule that inhibits the α4β7 receptor. The phase 2a open-label study evaluated the safety, tolerability, pharmacokinetics, and efficacy of MORF-057 in patients with moderately to severe UC. Thirty-five patients received 100 mg twice daily for 12 weeks, with 89% completing the induction period. The primary endpoint, a reduction in the Robarts Histology Index (RHI), was met with a significant decrease of −6.4 points (*p* = 0.0019). Additionally, 25.7% of patients achieved clinical remission, and 45.7% saw a clinical response. Pharmacokinetic analysis confirmed >99% receptor occupancy at week 12. The treatment was well tolerated, with low rates of treatment-emergent adverse events, primarily UC exacerbation and anemia. Ongoing trials are underway to further assess efficacy and determine optimal dosing [69].

### 4.5. Oral IL-23 Receptor Blocker

*JNJ-2113* is a first-in-class orally administered peptide that has been specifically designed to inhibit the IL-23 receptor, a key regulator in the activation of T cells in patients suffering from moderate-to-severe dermatological, rheumatological, and gastroenterological conditions driven by IL-23. Recent research has established the pharmacodynamic efficacy of JNJ-2110 through the use of both in vitro and ex vivo models involving rat colon tissue, as well as in vitro studies utilizing human colon explants and biopsies obtained from healthy volunteers. Similarly, JNJ-2113 has demonstrated in vitro activity in human colon explants. These findings are of great significance in advancing JNJ-2113 as a potential therapeutic agent for the treatment of IBD [70].

Following the demonstration of a notable impact with this agent in adults with moderate-to-severe plaque psoriasis, a phase 2 study was initiated to assess the safety and efficacy of JNJ-2113 in comparison with a placebo in patients with moderate-to-severe UC [71].

### 4.6. Oral TNF Agents

*TL1A* is a member of the tumor necrosis factor (TNF) family. TL1A interacts with its receptor, death receptor 3 (DR3), thereby influencing a number of cell lineages. ABX464, a prototype of TL1A, is an oral small molecule that modulates a specific microRNA, leading to the downregulation of proinflammatory cytokines and TH17+ cells [44]. In a phase 2b trial involving 254 patients with UC, ABX464 demonstrated significant efficacy in inducing clinical remission at daily doses of 25 mg, 50 mg, and 100 mg. These results were achieved without any major adverse events, and the drug demonstrated superior efficacy compared to a placebo [72,73].

A phase 2b trial was conducted to assess the efficacy and safety of *ABX464* = a small molecule that upregulates miR-124 in immune cells, in patients with moderate-to-severe UC. The study involved 254 patients, who were assigned to receive one of three doses of ABX464 (100 mg, 50 mg, or 25 mg) or a placebo. The results at week 8 demonstrated a notable improvement in the Modified Mayo Score in all ABX464 groups in comparison to the placebo, thereby indicating the efficacy of the drug in reducing disease severity. Furthermore, the rates of clinical response, clinical remission, and endoscopic improvement were higher in the ABX464 groups. The most commonly reported adverse effects were headaches, with dose-dependent adverse effects. The long-term 48-week open-label extension demonstrated sustained or improved efficacy, supporting the potential of ABX464 as a therapeutic option for patients with UC [44,73].

Verification of TL1A’s antifibrotic effects in human trials could prove particularly beneficial for patients with CD and other conditions. GSK298772, a promising oral small molecule targeting TL1A, has shown very encouraging results in a recent study, particularly in terms of safety and dosing, with further research on this agent ongoing [74].

It is noteworthy that a number of crucial parenteral TL1A agents are currently in development. The preliminary investigations into the parenteral TL1A agents demonstrated their efficacy in IBD patients, with only minor adverse effects. Furthermore, these agents have been shown to possess genetic biomarkers and may also reduce the formation of strictures by decreasing fibroblast activity and the deposition of collagen [75].

*RVT-3101* is a monoclonal antibody that targets TL1A, preventing its interaction with DR3 and thereby reducing inflammation. In a phase 2a trial, RVT-3101 demonstrated notable improvements in clinical remission and endoscopic healing in patients with UC, with a favorable safety profile [76]. The drug is currently being investigated further in phase 2b/3 trials for both UC and CD.

*PRA-023* is a TL1A inhibitor that competitively inhibits the TL1A/DR3 interaction, thereby reducing inflammatory activity in the gut. PRA-025 is currently in the early stages of clinical development, with preclinical studies indicating robust anti-inflammatory activity. Phase I clinical trials are currently being conducted to evaluate the safety and efficacy of the drug in patients with moderate-to-severe UC and CD [77].

It is our contention that oral small molecule agents will play a significant role in the management of patients with moderate-to-severe IBD. These FDA approved or investigational promising oral agents offer several advantages over traditional biologics, including the convenience of oral administration, their rapid onset of action, and the potential for more targeted control of inflammation with fewer adverse effects. Furthermore, these agents offer flexibility in combination with other therapies, rendering them suitable for the management of complex severe cases of IBD. Furthermore, these agents are more efficacious in patients with marked hypoalbuminemia and exhibit minimal immunogenicity, rendering them appealing alternatives for long-term management, as detailed in Table 5.

## 5. Limitations

While this paper provides an overview of the current therapeutic landscape for moderate-to-severe IBD, several limitations should be acknowledged. First, the rapidly evolving field of IBD treatment means that new therapies and evidence emerge frequently; thus, some of the information presented here may soon be supplemented by newer findings. Additionally, although we discuss various therapeutic options, the response to IBD treatments remains highly individualized, with variability influenced by genetic, environmental, and microbiome-related factors that are beyond the scope of this review. Furthermore, while we highlight the promise of novel treatments such as emerging oral therapies, the long-term safety and efficacy of these options require further research and validation in large, diverse patient populations. Finally, the focus of this paper on clinical management limits our discussion of economic factors, healthcare accessibility, and patient-reported outcomes, which are important components in achieving optimal patient-centered care in IBD management.

## 6. Conclusions

The management of moderate-to-severe inflammatory bowel disease has transformed significantly over the past few decades, with advancements in targeted therapies and a more personalized approach to treatment. Current strategies focus not only on alleviating symptoms but also on achieving sustained clinical remission, endoscopic/mucosal healing, and enhanced patient quality of life. The availability of biologics, small molecule therapies, and the promise of novel treatments offer a diverse array of options for clinicians to tailor therapy to individual patient needs. Despite these advancements, achieving optimal disease control remains challenging, and further research is needed to refine treatment protocols, improve long-term outcomes, and reduce the burden of inflammatory bowel disease on patients’ lives. The future of inflammatory bowel disease management lies in continued innovation, addressing unmet needs, and ensuring accessibility to effective therapies, ultimately working toward transforming inflammatory bowel disease from a debilitating condition to a manageable one.

## Figures and Tables

**Table 1:** Historical timeline of IBD treatments.

Year	Treatment
1979	Steroids, Sulfasalazine
1980	Antibiotics, Azathioprine, 6-MP
1993	5-ASA
1994	Budesonide
1995	Methotrexate
1998	Infliximab
2007	Second-generation anti-TNF agents
2014	New biologic agents
2015	Biosimilars
2019	Oral immunomodulators

**Table 2:** Criteria for the top-down approach in IBD.

Criteria	Rationale
Younger patients (<30 years) and early disease	Younger patients often have a more aggressive disease course and may benefit from early intensive therapy.
Significant hypoalbuminemia and persistent anemia	Hypoalbuminemia and anemia are markers of severe inflammation and poor prognosis, warranting more aggressive treatment.
Extensive anatomic involvement	Patients with extensive disease (e.g., pancolitis in UC or ileocolonic involvement in CD) are at higher risk for complications and may require early biologic therapy.
Significant extraintestinal manifestations (EIMs)	EIMs such as arthritis, uveitis, or pyoderma gangrenosum may indicate a more systemic inflammatory response, justifying early biologic use.
Severe anorectal disease	Patients with severe anorectal CD, including fistulas, abscesses, or strictures, often require aggressive treatment to prevent complications.
Deep and extensive ulcerations	Endoscopic findings of deep and extensive ulcerations are indicative of severe disease and warrant early biologic therapy.
Stricturing and/or penetrating CD	Patients with stricturing or penetrating disease are at high risk for complications, including obstruction and fistula formation, and may benefit from early intervention.
Prior surgical interventions	Patients who have already undergone surgery for IBD are at risk for recurrence and may benefit from early biologic therapy to prevent further complications.
Family history of severe CD	A family history of severe CD may suggest a more aggressive disease course, prompting earlier intervention.
Heavy smokers	Smoking is a well-known risk factor for more severe CD, and smokers may benefit from more aggressive treatment.

**Table 3:** FDA-approved IBD therapeutic options in 2024.

Category	Agents
**Anti-TNF Agents**	Infliximab for UC and CD, Adalimumab for UC and CD, Certolizumab for CD, Golimumab for UC
**Biosimilars**	Biosimilars for UC and CD
**Integrin Inhibitors**	Vedolizumab for UC and CD, Natalizumab for CD
**Anti-IL 12/23 Agents**	Ustekinumab for UC and CD
**Anti-IL-23 Agents**	Risankizumab for CD and UC, Mirikizumab for UC, Guselkumab for UC
**Oral Immunomodulators**	Tofacitinib for UC, Ozanimod for UC, Upadacitinib for UC and CD, Etrasimod for UC

**Table 4:** Common disease modifiers and recommended first-line therapy options.

Disease Modifier	First Treatment Choice	Reason
IBD and Pregnancy	Anti-TNFs, Vedolizumab	Fewer Adverse Effects: These drugs have a well-established safety profile during pregnancy.
IBD in Elderly Patients (>60 y/o)	Vedolizumab, Ustekinumab, Anti-IL-23s	Lower Infection Risk: These agents have a favoralesafety profile, particularly in reducing infection risks.
IBD and Malignancy History (e.g., Lymphoma)	Vedolizumab, Ustekinumab, Anti-IL-23s	Safer Profile: These agents are associated with a lower risk of malignancy compared to TNF inhibitors.
IBD and Psoriasis	Ustekinumab	Combined Effect: Effective for both IBD and psoriasis, making it a dual-purpose treatment.
IBD and Multiple Sclerosis (MS)	Ozanimod	Effective in treating both conditions.
IBD and Low Albumin Levels	Tofacitinib, Upadacitinib, Ozanimod, Etrasimod	on-alb-dependent: These therapies are effective without needing albumin for drug transport.
CD with Perianal Involvement	Anti-TNF (e.g., Infliximab, Adalimumab), Ustekinumab	Best Studied: Anti-TNFs have the most evidence supporting their efficacy in treating perianal CD.

**Table 5:** Advantages of oral small molecule agents in the management of patients with IBD.

Ease oral intake
Comparatively much cheaper
Predictable pharmacokinetic studies
Durable effectiveness comparable with biologics
Fast-on action and fast-off outcome
No immunogenicity
Effective in IBD patients with significant hypoalbuminemia
Potential for combination treatment with biologic agents

## Data Availability

Not applicable.
